# Comfortable, high-efficiency heat pump with desiccant-coated, water-sorbing heat exchangers

**DOI:** 10.1038/srep40437

**Published:** 2017-01-12

**Authors:** Y. D. Tu, R. Z. Wang, T. S. Ge, X. Zheng

**Affiliations:** 1 Institute of Refrigeration and Cryogenics, Shanghai Jiao Tong University, Shanghai 200240, China

## Abstract

Comfortable, efficient, and affordable heating, ventilation, and air conditioning systems in buildings are highly desirable due to the demands of energy efficiency and environmental friendliness. Traditional vapor-compression air conditioners exhibit a lower coefficient of performance (COP) (typically 2.8–3.8) owing to the cooling-based dehumidification methods that handle both sensible and latent loads together. Temperature- and humidity-independent control or desiccant systems have been proposed to overcome these challenges; however, the COP of current desiccant systems is quite small and additional heat sources are usually needed. Here, we report on a desiccant-enhanced, direct expansion heat pump based on a water-sorbing heat exchanger with a desiccant coating that exhibits an ultrahigh COP value of more than 7 without sacrificing any comfort or compactness. The pump’s efficiency is doubled compared to that of pumps currently used in conventional room air conditioners, which is a revolutionary HVAC breakthrough. Our proposed water-sorbing heat exchanger can independently handle sensible and latent loads at the same time. The desiccants adsorb moisture almost isothermally and can be regenerated by condensation heat. This new approach opens up the possibility of achieving ultrahigh efficiency for a broad range of temperature- and humidity-control applications.

Dramatically improving energy efficiency with adequate humidity control and sufficient fresh air ventilation is a long-standing focus of heating, ventilation, and air conditioning (HVAC) systems^1^ because electric HVAC systems are significant contributors to energy shortages and environmental problems^2^,^3^,^4^, generally requiring a series of compromises among energy efficiency, thermal comfort, and utility cost. Although adequate fresh-air ventilation is necessary to ensure indoor air quality, increasing air exchange will decrease the sensible heat ratio (SHR, defined as sensible load divided by the total cooling load; air-conditioning loads typically contain 60% sensible load for cooling and 40% latent load for dehumidification). With a reduction in SHR, a traditional vapor-compression air conditioner must be operated at a lower evaporation temperature (5–7 °C), which adopts a cooling-based dehumidification method to handle both sensible and latent loads together. This results in lower cooling capacity and a lower COP (2.8–3.8, in general), and a reheating process is required to meet supply-air requirements when the SHR drops below 0.6 (which is typical of Summer nights and swing seasons when sensible gains are low)^5^.

To solve this problem, considerable efforts have been made to develop efficient alternative refrigeration systems such as caloric-based technologies^6^,^7^,^8^,^9^ (those using the magnetocaloric, elastocaloric, barocaloric, or electrocaloric effects) and innovative system designs such as temperature- and humidity-independent control^10^ (THIC). Although caloric-based technologies show great potential for energy savings, they are still far from being practically applied^11^. A THIC system usually consists of a thermally driven dehumidification unit (for example, a liquid or solid desiccant system) and a vapor-compression air conditioner specializing in handling the sensible heat load (such as variable-refrigerant flow technology). It was reported that THIC systems might save 25–50% of electrical consumption^10^,^12^,^13^,^14^ by adopting a higher evaporation temperature (e.g. 15–20 °C), and the corresponding electric COP increases by about 40–60% (refs 10 and 12) under different operating conditions compared to conventional HVAC systems. However, the COP (equal to latent load divided by primary thermal energy input) of current solid-desiccant systems is quite small, typically ranging from 0.5 to 1.0 (ref. 15) due to high regeneration temperature [usually in the range 60–120 °C (refs 15 and 16)]. Even though solar heat or industrial waste heat can sometimes be used, a large volume and high utility cost limits the large-scale application of solid-desiccant systems in residential buildings^12^.

It is quite natural to consider whether desiccant dehumidification could be used in an isothermal process, since, if adsorption heat could be easily taken away, then desiccant dehumidification will be very efficient, while desiccant regeneration can be handled by the waste heat from the air-conditioning system, for example, as condensation heat. In this case, it would be reasonable to have an evaporation temperature of 15 °C for sensible cooling and dehumidification and a condensation temperature of 45 °C for desiccant regeneration^12^. Then, a novel desiccant material having a sufficient water adsorption capacity difference under two-cycle conditions [such as 15 °C/80% relative humidity (RH) and 45 °C/30% RH] should be adopted. It is estimated that if the HVAC systems have 40–50% latent heat the evaporation temperature can increase from approximately 5–7 to approximately 15–17 °C and the condensation temperature can decrease from approximately 50–55 to approximately 40–45 °C, and then the COP can be nearly doubled.

Here, we report a novel concept for a desiccant-enhanced direct expansion heat pump (DDX HP) (Fig. 1a) based on the proposed water-absorbing heat exchanger (WSHE) fabricated by coating a desiccant on the surfaces of a conventional evaporator and condenser. In order to guarantee continuous operation, two of the same WSHEs will switch from evaporator (condenser) into condenser (evaporator) alternately (Fig. 1b). The sensible load is handled in the same way as before, by convection, but without overcooling (an evaporation temperature approximately 15–17 °C), and the desiccant coatings treat the latent load in a nearly isothermal way (Fig. 1c). Therefore, the processed air leaving the evaporator satisfies the requirement for supply air (Fig. 1d). In this DDX HP, the evaporation temperature rises while the condensation temperature drops compared to traditional air conditioners, because a WSHE does not need to cool the process air below its dew point to condense the moisture, and the adsorbed water evaporation strengthens the heat-dissipation capacity of the condenser. Therefore, a DDX HP shows a great potential of achieving much higher energy efficiency.

In fact, similar concepts of adsorption heat exchangers have been studied by several researchers^13^,^17^,^18^,^19^,^20^, but most were designed to dehumidify the air or to be used for adsorption refrigeration that focused on the mass-exchange process. To achieve the above-mentioned goals, four problems need to be solved: (1) novel desiccants that have an adequate moisture uptake capacity difference under two-cycle conditions should be invented; (2) sensible heat transfer should not be obviously weakened by the desiccant coated layer; (3) a matched control strategy with a DDX HP to achieve high efficiency at any mix of sensible and latent loads should be developed; and (4) the loss of sensible-load capacity due to operational mode switching should be minimized. This is the first time, to our knowledge, that a high-efficiency approach has been devised that inherits advantages of THIC, but also has the merits of compactness, low cost, and realization of the effective utilization of condensation heat.

## Results

### Desiccant-coated layer

**Figure 1:**
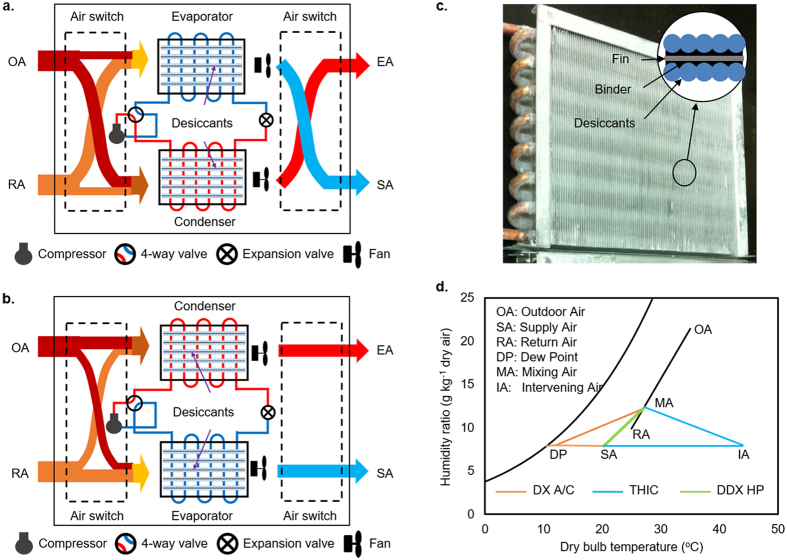
Schematic of the structure and operational modes of a novel concept for a desiccant-enhanced DX heat pump. (**a**) Representative structure of a desiccant-enhanced DX heat pump consisting of one compressor, one four-way valve, one expansion valve, two water-sorbing heat exchangers (WSHE), two fans, and two air switches; all of the components are located in a compact package. In the first operational mode, the top WSHE works as an evaporator and the bottom WSHE works as a condenser, with the supply air mixed with outdoor air (OA) and return air (RA) directed into the top WSHE by the front air switch and finally supplied to the room by the back air switch. The exhaust air mixed with OA and RA is directed into the bottom WSHE by the front air switch and is finally discharged to ambient air by the back air switch. (**b**) Second operational mode in which the four-way valve reverses the refrigerant flow in the two WSHEs, the top WSHE works as an evaporator and the bottom WSHE works as a condenser, the supply air is directed into the top WSHE by the front air switch, and finally supplied to the room by the back air switch. The exhaust air is directed into the top WSHE by the front air switch and is finally discharged to ambient air by the back air switch. (**c**) Photograph of a WSHE. The inset represents the structure of the desiccant-coated fin. (**d**) Air-processing line of the proposed heat pump for cooling and dehumidification compared to that of a DX air conditioner and THIC system, which directly satisfies the supply air with no overcooling or reheating. The sensible load is handled in the same way as before, by convection, while coated desiccants treat the latent load in a nearly isothermal way.

Our concept of a water-adsorbing heat exchanger is illustrated in Fig. 1c, which is typically operated at 10–20 °C for cooling/adsorption and at 40–50 °C for heating/desorption. In this design, a desiccant-coated layer is adhered onto the fin surface of the base heat exchanger (here we use a fin-and-tube heat exchanger as an example) to form an integrated heat and mass exchanger. The desiccant-coated layer is based on a composite mesoporous, silica-gel-supported lithium chloride (CSGL) desiccant made from lithium chloride as the filler, owing to the appropriate deliquescence relative humidity and mesoporous silica-gel particles (50–100 mesh) as the matrix due to a large void volume. Such a composite desiccant can provide a small adsorption capacity at low relative humidity (less than 50% RH) and high adsorption capacity at intermediate relative humidity (in the range 50–80% RH) due to the combination of capillary condensation and solution absorption. Given that the evaporation temperature required for cooling often approaches the dew point and that the relative humidity on the fin surface of the condenser is generally below 30% RH, CSGL perfectly satisfies the requirement of a sufficient water adsorption capacity difference at two typical cycle conditions (for example, 15 °C/80% RH and 45 °C/30% RH). In addition, the water-borne compound adhesive used as a binder (approximately a particle radius thick) not only guarantees strong adhesion of granular desiccants to the coil, but also provides a protective coating to prevent chloride corrosion. Meanwhile, it is easier to avoid corrosion or carryover of liquid droplets by controlling the salt content^21^ and to prevent saturation or leakage by adjusting the duration of the moisture uptake process. In fact, the thickness of the desiccant-coated layer is the one important parameter that affects both the desiccant loading and the convection heat transfer. The optimal value that satisfies both criteria is approximately 10% of the fin space based on our experience. Details of the WSHE fabrication are given in the Methods section.

**Figure 2:**
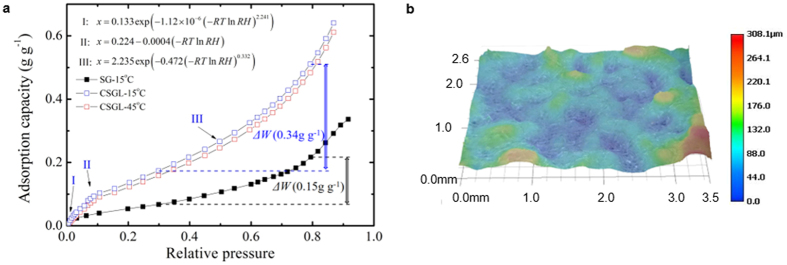
Isotherms of CSGL and digital microscopy photograph of the desiccant-coated layer surface. (**a**) Water-vapor adsorptive isotherm of CSGL at 15 and 45 °C compared to pure mesoporous silica gel. (**b**) Layer-thickness illustration from digital holographic microscopy (DHM).

As a model system, the salt content of CSGL is 16.2 wt.%, and its isotherms are presented in Fig. 2a. It can be seen that the water content of CSGL at 15 and 45 °C under a specific relative humidity shows little difference, indicating that the moisture uptake capacity of CSGL is insensitive to temperature but mainly depends on the relative humidity. The adsorption capacity difference between a typical cooling/adsorption phase (80% RH) and a condensation/desorption phase (30% RH) is 0.34 kg kg^−1^, more than twice that of silica gel. The surface morphology looks similar to shattered glass and the globular silica gels are embedded in a solidified silica sol (Supplementary Fig. 1). Its thickness is estimated to be in the range 0.2–0.25 mm derived from the desiccant loading on unit area, 158.6 g m^−2^, considering that the density of 50~100 mesh granular silica-gel is 600~700 kg m^−3^. The altitude difference of the desiccant layer is about 0.05–0.15 mm indicated by variations of color shown in Fig. 2b, which also confirms the distribution of globular silica gels. The pore-size distributions of the desiccant-coated layer show double peaks around the points at 4 and 20 nm (Supplementary Fig. 2), with an average pore diameter 8.4 nm which is slightly smaller than that of mesoporous silica gel (10.4 nm). A rational assumption would be that large pores are partially filled or at least narrowed. The cumulative pore volume at 4 nm is very small, while that at 20 nm is relatively larger, which represents the solidified binder and globular silica gel, respectively. The specific surface area and pore volume are 143 m^2^ g^−1^ and 0.25 cm^3^ g^−1^, respectively. Other textural properties of CSGL film, such as thermal conductivity, specific heat capacity, and adsorption rate constant, were also measured as 5.27 W m^−1^ K^−1^, 1.2 kJ kg^−1^ K^−1^, and 1.0 × 10^−3^ s^−1^, respectively.

### Weakly coupled heat and mass transfer

**Figure 3:**
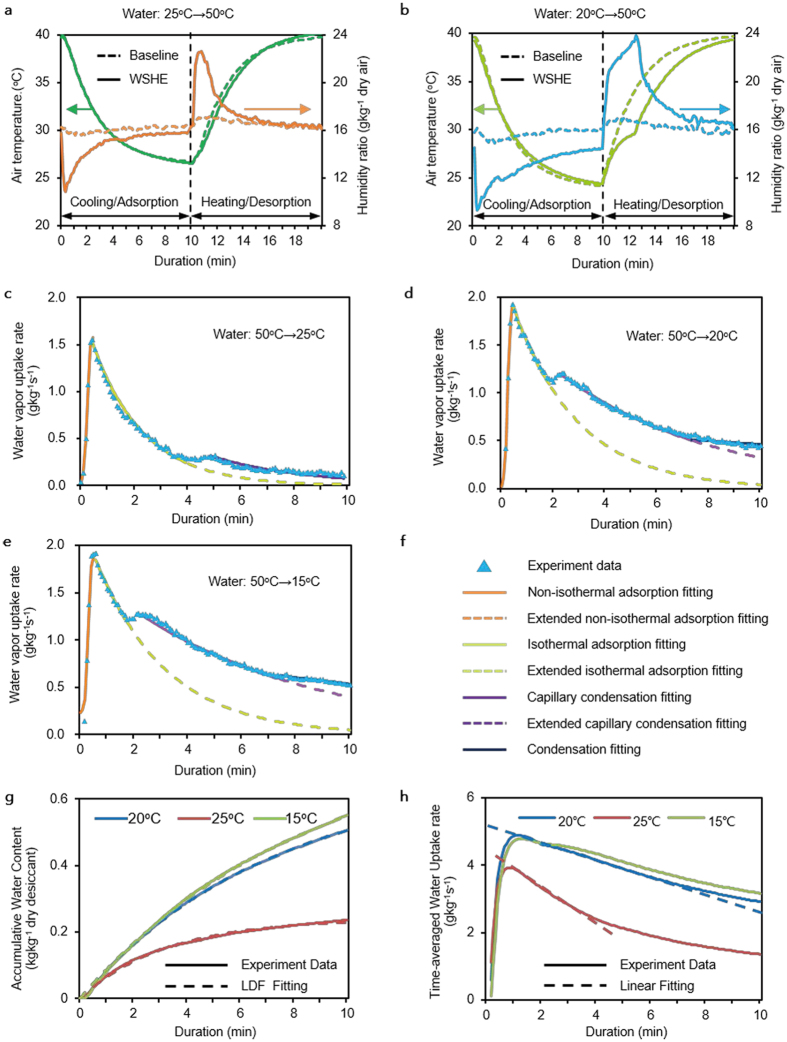
Airside characteristics of heat and mass transfer of the WSHE. (**a**) Outlet air dry bulb temperature and humidity ratio of the WSHE and the baseline at 50 °C hot water and 25 °C cold water. (**b**) Outlet air dry bulb temperature and humidity ratio of both heat exchangers at 50 °C hot water and 20 °C cold water. (**c–e**) Water vapor uptake rates and the mechanisms of water vapor uptake at different cold water temperatures. (**f**) Legend used for (**c–e)**. (**g**) Experimental results and linear driving force model (LDF) fitting of accumulated water content over time at different cold water temperatures. (**h**) Experimental data and linear fitting of the time-averaged water uptake rate.

A desiccant-coated layer generates additional thermal resistance for conduction but decreases the thermal resistance for convection owing to the enhancement of process air velocity flowing across the WSHE due to the narrowed fin space. Meanwhile, instead of condensing the moisture by overcooling the air as a cooling-based dehumidifier does, a desiccant-coated layer attracts moisture from the air by creating an area of low vapor pressure at the surface of the desiccant. Therefore, the WSHE shows some special heat- and mass-transfer behaviors different from the well-known features of conventional heat exchangers. The experimental setup for characterization of heat- and mass-transfer behaviors (and their efficiencies) is shown in Supplementary Fig. 3 and detailed descriptions of the WSHE are illustrated in Supplementary Table 1. For convenience, we chose liquid water as the heat-transfer media. The test conditions are illustrated in Table 1 and the test instruments are described in Supplementary Note 1. Each test procedure includes two processes: the cooling/adsorption process driven by cold water and the heating/desorption process driven by hot water. These two processes operate interchangeably and the dynamic behaviors of heat and mass transfer are measured by recording the change of the dry bulb temperatures as well as the humidity ratios of inlet and outlet air as a function of time. The results are compared with those from a baseline heat exchanger of the same size, as shown in Fig. 3a and b, and more detailed experimental data are presented in an extended data sheet.

The convective heat- and mass-transfer coefficients for the WSHE are kept unchanged compared to the baseline. As shown by the cooling/adsorption process depicted in Fig. 3a, the exit dry bulb temperatures of both the baseline and WSHE, *T*_*a*,*eo*_, are basically the same. This indicates that the desiccant-coated layer barely alters the sensible load capacity. It is reasonable to infer that the desiccant-coated layer induces subtle changes in the convective heat-transfer coefficient *h*, or, in other words, the heat-transfer resistance imposed by conduction across the desiccant-coated layer is relatively small. Similarly, the convective mass-transfer coefficient *h*_*m*_ is neither strengthened nor weakened according to the heat- and mass-transfer analogy for a fin-and-tube heat exchanger because both the heat and mass transfer are limited by (heat) conduction and (water vapor) diffusion, respectively, through the same laminar boundary layer covering the fin surface^22^.

The driving force of heat transfer for the WSHE is almost identical to that of the baseline, while the driving force of mass transfer can be dramatically improved. The exit dry bulb temperatures in Fig. 3a and b indicate that the driving force of heat transfer, Δ*T*, is slightly weakened, which depends on the exit humidity ratio difference between the two heat exchangers, Δ*d*. A rational explanation is that a larger Δ*d* results in more adsorption/desorption heat accumulated in/consumed by the desiccant-coated layer, and the temperature fall/rise of the desiccant-coated layer is retarded. The exit humidity ratios shown in Fig. 3a and b clearly reveal that an obvious improvement in the mass-transfer driving force can be easily achieved. It is worth noting that when the inlet cold water temperature *T*_*w*_ is above the moisture’s dew point *T*_*dp*_, the latent load capacity of the baseline can be almost ignored, while that of the WSHE is still remarkable, as shown in Fig. 3a. When *T*_*w*_ approaches *T*_*dp*_, the advantages of dehumidification by the WSHE become more prominent.

The desiccant-coated layer shows a high adsorption capacity due to capillary condensation and a fast adsorptive dynamic, which is confirmed by the isothermal adsorption experiments on the original desiccant. As shown in Fig. 3c, when *T*_*w*_ is above *T*_*dp*_ the entire water uptake process sequentially experiences three different water uptake modes: non-isothermal adsorption, isothermal adsorption, and capillary condensation. Once *T*_*w*_ approaches *T*_*dp*_ (Fig. 3d), cooling condensation occurs. However, if *T*_*w*_ is lower than *T*_*dp*_ (Fig. 3e), the dynamic water uptake behaviors are almost equivalent to the former. These results clearly demonstrate that capillary condensation contributes almost two-thirds of the adsorption capacity before cooling condensation occurs. Although there are varieties of uptake modes, the water content Δ*w* at varied time *t* and different *T*_*w*_ generally agree with the LDF model^23^,^24^ [linear driving force 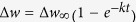] very well, as shown in Fig. 3g. The only exception appears in the initial non-isothermal adsorption stage (duration less than 15 s and Δw less than 5% of the total). This indicates that the entire adsorption process can be viewed as isothermal. The time-averaged water uptake rates (ratios of water content to duration) 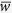 further prove this feature (Fig. 3h). Although 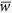 increases rapidly at the initial stage, it begins to decrease in an approximately linear way with the passage of time. The LDF fitting results show that the adsorptive rate constant *k* is enhanced from 1.76 × 10^−3^ to 2.32 × 10^−3^/s^25^, with 31.82% improvement with respect to the value measured in the isothermal adsorption experiments for the original desiccant, as shown in Table 2. In addition, the values of *k* decrease when *T*_*w*_ becomes lower, but at a slighter decreasing rate (Table 2).

In short, the WSHE shows a beneficial airside characteristic of weak coupling of heat and mass transfer. More concretely, the sensible load capacity is the same as that of baseline depending on the temperature difference between the processing air and the WSHE, while the latent load capacity can be enhanced dramatically relying on not only heat-exchanger temperature but also on the duration of the cooling/adsorption process. Taking advantage of this additional parameter compared to conventional heat exchangers, the exit humidity ratio can be easily controlled by adjusting the duration without having to change the heat-exchanger temperature.

This feature presents a great opportunity to achieve temperature- and humidity-independent control by using a single heat exchanger. Moreover, for a WSHE in a vapor compression cycle, the evaporation temperature will increase significantly (e.g., from 5 to 15 °C) resulting from the omission of deep cooling for dehumidification. The condensation temperature will effectively decrease (e.g., from 50 to 40 °C) owing to the enhancement of airside heat dissipation due to adsorbed water evaporation. Moreover, for a room air-conditioning system, the latent heat can be treated purely by a desiccant and the desiccant will be regenerated by using condensation heat, and the release of condensation heat to the ambient will be dramatically reduced.

### Temperature and humidity loosely coupled control

In this paper, we propose a novel concept of temperature and humidity loosely coupled control (THLC) in a single-heat-pump package according to the characteristics of loosely coupled heat and mass transfer of a WSHE as analyzed above. The basic idea of this novel concept is that the sensible and latent loads can be treated independently of a WSHE. According to the ε − *NTU* method^26^,^27^, the sensible heat capacity of a WSHE as an evaporator, *Q*_*c*,*s*_, can be estimated, ignoring heat capacity loss due to its thermal inertia,


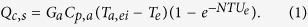


According to water mass balance, its latent heat capacity, *Q*_*c*,*l*_, can be obtained as


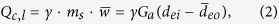


where *G*_*a*_ is the supply air flow in kg s^−1^ *m*_*d*_ the desiccant mass in kg, *T*_*a*,*ei*_ the inlet air temperature in °C, *T*_*e*_ the evaporation temperature in °C, *C*_*p*,*a*_ the specific heat of air, *NTU*_*e*_ the number of heat transfer units, *d*_*ei*_ and 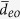 the dry air inlet and time-averaged outlet humidity ratios in kg kg^−1^, respectively, *γ* the heat of condensation in kJ kg^−1^, and 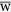 is the time-averaged water uptake rate in kg kg^−1^ s^−1^.

The design and control strategies of this novel concept are, firstly, that the required supply air temperature is adjusted by controlling the evaporation temperature illustrated by Eq. (1), and, secondly, that the supply air humidity 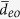 is satisfied by adjusting the adsorption/desorption duration according to the curves of the time-averaged water uptake rate of the desiccant layer at a specific temperature, as shown in Fig. 3h and illustrated by Eq. (2). For example, as shown in Fig. 3h, when the latent load becomes larger while the sensible load remains unchanged, only shortening the duration and keeping the evaporator temperature unchanged are needed. In contrast, when the latent load remains unchanged while the sensible load is increased, a reduction in the evaporation temperature and an extension of the duration are necessary. In this way, varied sensible and latent loads of buildings in any climate can be flexibly accommodated. Therefore, a WSHE with THLC can effectively improve the load capacity and energy conversation of a heat pump as well as control flexibility. At the same time, this concept keeps the entire system compact and simple.

### Energy efficiency

**Figure 4:**
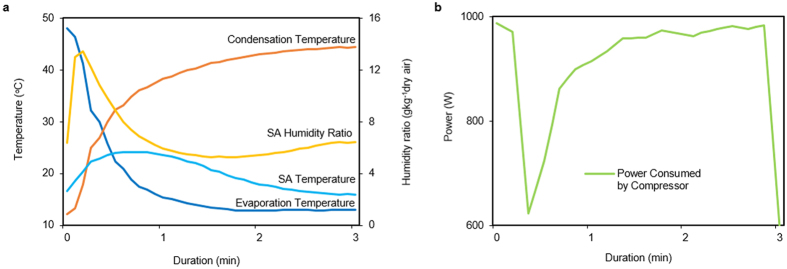
Energy performance of desiccant-enhanced DX heat pump. (**a**) Evaporation temperature, condensation temperature, supply air dry bulb temperature (SA temperature.), and humidity ratio (SA humidity ratio) of the demonstration setup with 3-min duration. (**b**) Power consumed by compressor.

For the first demonstration of this approach, we developed a single packaged desiccant-enhanced DX heat pump including two parts, a vapor compression loop and air ducts, both of which are located in a cabinet with two air inlets (outdoor air, OA, and return air, RA) and two air outlets (supply air, SA, and exhaust air, EA) (Fig. 1a and b). Two identical WSHEs in a vapor compression loop work as an evaporator or condenser interchangeably with the assistance of a four-way valve. Correspondingly, the processing air (mix of OA and RA) and the exhaust air (mix of OA and RA) can be switched to the evaporator and condenser by using the front duct air switch. The outlet air of the evaporator and condenser can be directed to SA and EA by utilizing the back duct air switch. Details of system design are given in Supplementary Fig. 4 and Supplementary Table 2 and the test instruments are described in Supplementary Note 1. The performance of the demonstration has been tested under a common scenario in Summer for room air conditioning (outdoor 35 °C/60% RH and indoor 25 °C/50% RH, 30% outdoor fresh air) per standard ISO 5151:1994. The experimental results (Fig. 4a and b) show that the condensation temperature approaches 43 °C, the evaporation temperature is approximately 13 °C, the average supply air dry bulb temperature is approximately 20 °C, the humidity ratio is approximately 8.5 g/kg dry air (50% RH), and the power consumed by the compressor and fans are roughly 980 and 149 W, respectively. In other words, the total cooling capacity is 7.0 kW with a compressor COP of approximately 7.14 and a system COP of 6.2. These are nearly double the values of current conventional room air conditioners.

## Discussions

**Figure 5:**
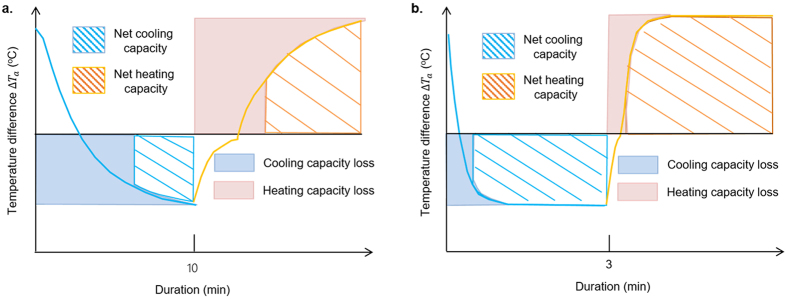
Effects of refrigerant on sensible load capacity loss due to mode switching. (**a**) Sensible heat load capacity loss compared to constant evaporation/condensation temperature with water as heat-transfer media. (**b**) Sensible heat load capacity loss compared to constant evaporation/condensation temperature with phase-change refrigerant as heat-transfer media.

The above results have shown the efficacy of a WSHE in improving the effectiveness and energy efficiency for cooling and dehumidification owing to the evaporation temperature increase and the condensation temperature decrease. By taking advantages of the weak coupling of heat and mass transfer, comfortable supply air and a significant reduction in energy consumption are achieved. However, in order to operate the system continuously, two WSHEs in a cycle are required to work alternatively as an evaporator and condenser. This frequent mode switching will inevitably introduce some sensible cooling loss of the processing air before the heat-exchanger temperature becomes steady (Fig. 5a), compared to a constant evaporation/condensation temperature. Moreover, the WSHE probably consumes more cooling capacity provided by heat-transfer media than the baseline owing to the WHSE’s thermal inertia, the charged heat-transfer media, and the large adsorption heat. These side effects are potentially harmful to the exit dry bulb temperature control and the energy efficiency of the WSHE, and, thus, how to acquire full functionality of the advantages and correct the deficiencies still needs further study.

According to the results of the experimental section of this paper, the temperature of a desiccant-coated layer plays a key role in sensible load capacity loss. If the WSHE temperature exhibits a step change at the initial stage of mode switching, it is reasonable to infer that the sensible load capacity loss is sufficiently small with respect to the total cooling capacity. In fact, there are several factors, such as flow pattern and the flow rates of refrigerant, heat of adsorption, tube-side heat-transfer coefficient, and total heat capacity of the WSHE, that can potentially affect the thermal response rate of the desiccant-coated layer. Therefore, it is essential to find out how each factor influences performance.

To achieve a better understanding, a series of experimental demonstrations of the heat-transfer performance of a WSHE with different desiccants and under different working conditions (such as different refrigerants and climates) are important and necessary; however, due to time constraints and limited capability to construct multiple setups, only a few experiments under typical conditions are implemented. Nevertheless, theoretical analysis of dynamic behaviors of heat and mass transfer suggests that a WSHE can effectively enhance heat and mass transfer with careful selection of refrigerant and desiccant according to the specific requirements of the applications. To further understand the effects of mode switching, we propose a simple but accurate model using the lumped parameter method (Supplementary Manuscript). Theoretically, the cooling/adsorption and heating/desorption phases are symmetric. However, desorption usually finishes earlier than adsorption in real-time experiments (Fig. 3a and b). As a result, the evaporator is revealed to be the bottleneck to improved cycle performance.

Based on this model, the approximate analytic solution of the outlet air temperature *T*_*a*,*eo*_ and the time-averaged outlet humidity ratio 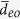 for an evaporator are obtained as


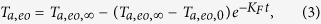



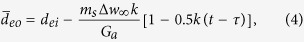


where 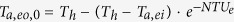 and 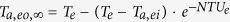.*T*_*a*,*eo*,∞_ represents the outlet air temperature under steady state in °C, *T*_*a*,*eo*,0_ the outlet air temperature at the initial state in °C, 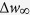 the largest possible adsorption capacity difference at a specific cycle condition in kg kg^−1^, *K*_*F*_ the fin temperature change rate constant in s^−1^, *k* the water uptake rate constant in s^−1^, *t* the duration of the total cooling/adsorption process in s, and τ the duration of the initial stage when the time-averaged water uptake rate reaches the maximum, in s.

The cooling load capacity loss rate of the WSHE, *ε*_*sl*_, can be expressed as the ratio of the real cooling load to the theoretical value under constant evaporation temperature,


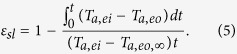


Substituting Eq. (3) into (5) yields


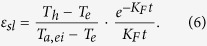


In this study, our assumed conditions were a steady-state evaporation temperature *T*_*e*_ of 15 °C, a steady-state condensing temperature *T*_*h*_ of 45 °C, an inlet air temperature *T*_*a*,*ei*_ in the range 27–35 °C, and a duration of adsorption of approximately 100–1000 s. According to Eq. (6), it is easy to find that when the value of *K*_*F*_ is larger than 0.02 s^−1^, the value of *ε*_*sl*_ will be less than 10% (Fig. 5b). These results can also be confirmed by experimental data fitting referred to in Supplementary Table 2, where the values of *K*_*F*_ are 5.60 × 10^−3^ and 6.01 × 10^−3^ s^−1^ with cold water at 20 and 25 °C, respectively. The corresponding values of *ε*_*sl*_ go beyond 50%, as shown in Fig. 5a. In fact, *K*_*F*_ comprises a number of factors, such as the evaporation temperature change rate constant *K*_*R*_, heat capacity of the WSHE, heat of adsorption, *q*_*st*_, and heat-transfer resistance by convection in the tube and airside. However, scale analysis shows that *K*_*R*_ and *q*_*st*_ have the greatest influence on *K*_*F*_. A large value of *K*_*R*_ due to heat transfer by phase change and a low value of *q*_*st*_ can result in a large value of *K*_*F*_. According to our experience, refrigerants with high flow velocity, low charge amount, low specific heat capacity, and high phase-change heat-transfer coefficient are of great interest. These merits are helpful to improving the thermal response to refrigerant temperature change.

## Conclusions

In summary, efficient cooling and dehumidification plays an important role in building energy conservation, especially for the goal of a net-zero-energy building. In this paper, we have analyzed the performance barriers of traditional heat-exchanger and desiccant systems for cooling and dehumidification. Moreover, a temperature and humidity loosely coupled control strategy based on a water-sorbing heat exchanger (WSHE) is proposed and applied so that the air leaving the evaporator rightly delivers the required supply air. The WSHE was fabricated by coating desiccants directly onto the surfaces of a traditional heat exchanger. By well controlling the thickness of the desiccant-coated layer, the WSHE achieved weakly coupled heat and mass transfer at the airside. The sensible heat transfer depends on evaporation (condensation) temperature, but mass transfer is affected by both evaporation (condensation) temperature and the duration of adsorption (desorption). As an evaporator, a WSHE handles the latent load through the desiccant-coated layer without overcooling. Therefore, the sensible load is reduced and treated, as before, by convection with no reheating. As a condenser, a WSHE can regenerate the desiccant-coated layer by condensation heat. As such, the difference between the evaporation temperature and the condensation temperature can dramatically decrease from approximately 50 °C to approximately 30 °C.

To demonstrate the advantages of a WSHE, a packaged desiccant-enhanced heat pump is designed and two same WSHEs are applied to replace the ordinary fin-tube coils in a traditional DX air conditioner. Experimental results showed that the system COP was 6.2 under typical Summer conditions per ISO 5151:1994. To our knowledge, this is the first time that an approach can offer sufficient temperature and humidity control and adequate fresh air ventilation in any climate with doubled energy efficiency compared to current room air conditioners, and, most importantly, without sacrificing cost and compactness.

## Methods

### Fabrication of water-adsorbing heat exchanger

We choose the fin-tube heat exchanger (Supplementary Tables 1 and 2) as an example. First, water-borne compound adhesive (Shanghai Maobang Co. Ltd) serving as a binder was coated on the coil surfaces by dip-coating^28^ at 25 °C at 1900–2600 cps. Mechanical swing technology was applied to form a smooth binder coating with a typical thickness of the desiccant’s granule radius. Second, mesoporous silica gel powders of 50–100 mesh (Shanghai Lingyi Co. Ltd) were adhered onto the coil surfaces via an electrostatic spraying method^29^ to a layer thickness of approximately 0.25 mm. The coil was dried and powders not clinging tenaciously enough to the coil were blown away with compressed air. In the following step, the coil was immersed into a 38 wt.% LiCl solution with ample time to assure total immersion. Finally, after drying, the coil was put into a climatic chamber under 30 °C/90% RH conditions in order to flush the overload of LiCl out of the desiccant. Another cycle of drying at 100 °C for 4–6 h was necessary to obtain the final WSHE.

### Characterizations of desiccant-coated layer

#### Measurement of thermal properties

The thermal conductivity and diffusivity of composite samples were measured using a xenon flash analyzer, which employs the principle of the laser-flash measuring method. In the experiments, a beam of light pulses was emitted to the bottom surface of a testing sample, leading to a temperature rise on the top surface. By analyzing the resulting temperature-time curve, the thermal diffusivity can be determined. Meanwhile, the specific heat can be obtained via means of a comparative method using a standard sample. Then, with the measured density, the thermal conductivity can be derived. The largest possible errors for the thermal diffusivity and specific heat from the instrument are 3% and 5%, respectively.

#### Measurement of adsorption kinetics

Adsorption kinetics measurements of composite samples were carried out in a constant-temperature and -humidity chamber, with a silica gel as a reference. The chamber can supply air with constant conditions. Deviations of temperature and relative humidity are ±0.5 °C and ±3%, respectively. The defined working conditions were set as 20 °C and 70% RH. Before the test, the samples were dried at 100 °C in an oven. The weights of the samples were recorded at set intervals by an electronic balance with an accuracy of 0.001 g.

#### Measurement of adsorption isotherms

Water adsorption isotherms of composite desiccant were measured using an improved ASAP2020 system with a water vapor generator, a jacketed beaker, and a constant-temperature water bath attached to it. The temperature of the sample tube is controlled by the constant-temperature water bath and the jacketed beaker. The accuracy of the water bath is ±0.05 K.

#### Measurement of texture properties

Texture properties, such as surface area, pore volume, and pore size were tested and analyzed using the ASAP2020 system. The system is based on the principle of the static volumetric technique and obtained nitrogen adsorption/desorption isotherms at 77 K. The surface area was calculated based on the BET equation and the pore-size distributions were obtained according to Barrett-Joyner-Halenda theory. The experimental error of the ASAP2020 apparatus was mainly caused by temperature and pressure transducers. The accuracy of the temperature transducer is ±0.02 K and the corresponding testing error of the pressure transducers is 0.1%.

## Additional Information

**How to cite this article**: Tu, Y. D. *et al*. Comfortable, high-efficiency heat pump with desiccant-coated, water-sorbing heat exchangers. *Sci. Rep.* **7**, 40437; doi: 10.1038/srep40437 (2017).

**Publisher's note:** Springer Nature remains neutral with regard to jurisdictional claims in published maps and institutional affiliations.

## Supplementary Material

Supplementary Information

Supplementary Extended Dataset

## Figures and Tables

**Table 1:** Test conditions for characterization of airside heat and mass transfer.

	Inlet water		Inlet air
	Cold	Hot	
Flowrate	0.050 kg s^−1^	0.057 kg s^−1^	0.08625 m^3^ s^−1^
Test 1	25 °C (>dew point)	50 °C	30 °C 60%RH
Test 2	20 °C (~dew point)		
Test 3	15 °C (<dew point)		

**Table 2:** Adsorptive dynamics shown in Fig. 3g fitted by the LDF model [Δ*w* = Δ*w*_∞_(1 − *e* ^−*kt*^)].

Conditions	Parameters	Values	Error (*R*^2^)
25 °C	Δ*W*_∞_/(kg kg^−1^)	0.23994	1.05 × 10^−3^
	*k*/(s^−1^)	5.15 × 10^−3^	6.00 × 10^−5^
20 °C	Δ*W*_∞_/(kg kg^−1^)	0.67217	3.43 × 10^−3^
	*k*/(s^−1^)	2.32 × 10^−3^	2.00 × 10^−5^
15 °C	Δ*W*_∞_/(kg kg^−1^)	0.79602	6.87 × 10^−3^
	*k*/(s^−1^)	1.95 × 10^−3^	3.00 × 10^−5^
Isothermal^25^ 20 °C/70% RH	Δ*W*_∞_/(kg kg^−1^)	0.28085	5.79 × 10^−3^
	*k*/(s^−1^)	1.76 × 10^−3^	9.72 × 10^−3^
