# Supplementary material for: Comfortable, high-efficiency heat pump with desiccant-coated, water-sorbing heat exchangers
Source: Sci Rep. 2017 Jan 12;7:40437. doi: 10.1038/srep40437 (PMC5227918; doi:10.1038/srep40437)
Supplement: Supplementary Information [file srep40437-s1.pdf]

Supplemental Information

For

Comfortable, high-efficiency heat pump with desiccant-coated, water-absorbing heat exchangers

by

Yaodong Tu, Ruzhu Wang, Tianshu Ge, Xu Zheng

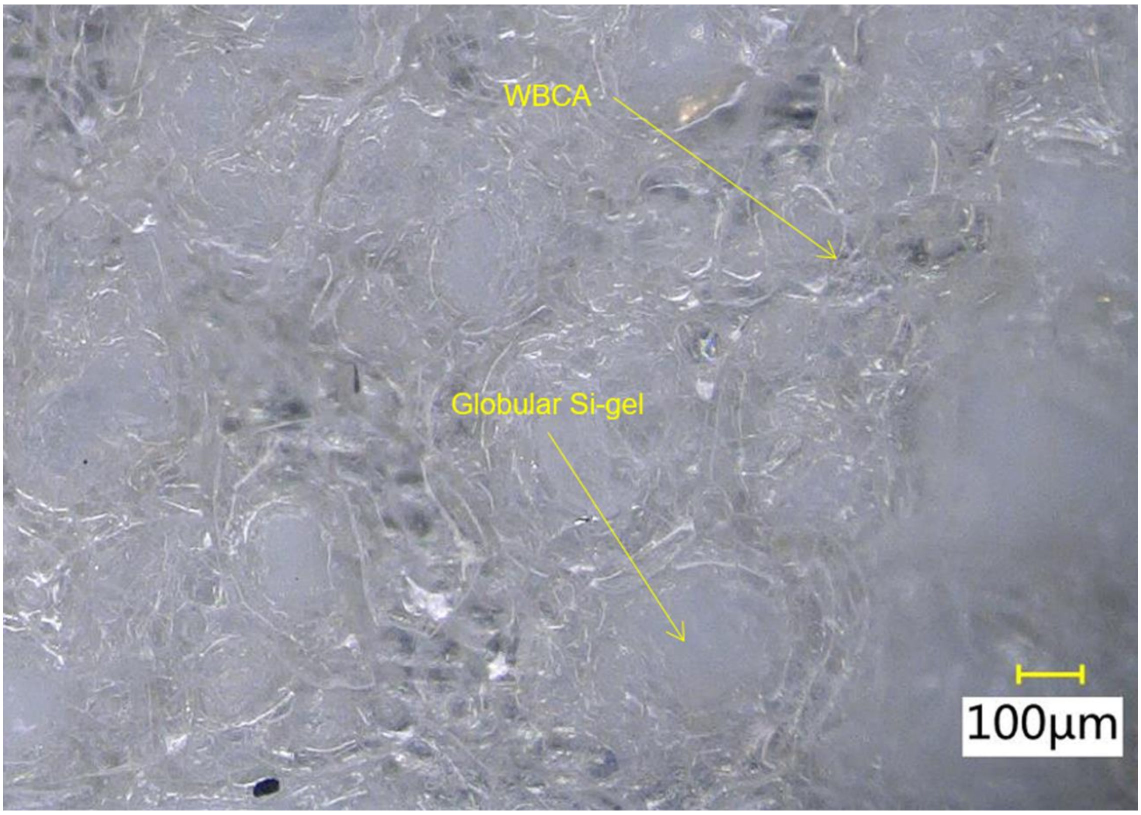

Fig. 1 Digital microscope surface morphology of the desiccant film.

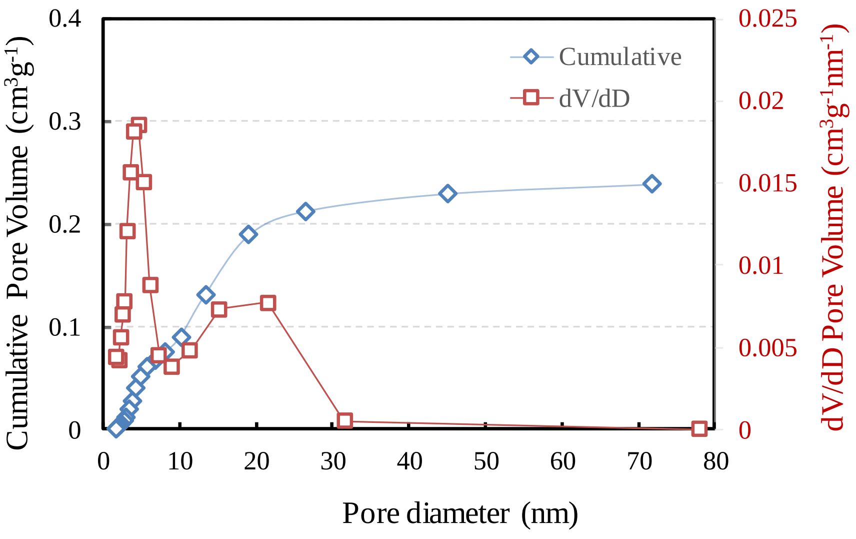

Fig. 2 Pore size distributions and cumulative pore volume of desiccant coated layer.

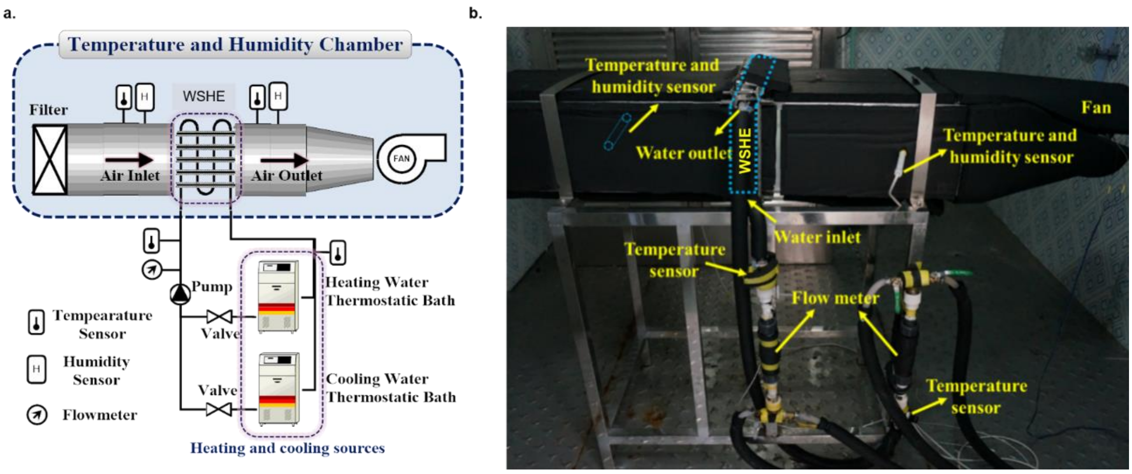

Fig. 3 Schematic diagram and photo of the experimental setup used to measure the dynamic behaviors of heat and mass transfer. One quadrate air channel with an area of 280mm×200mm is adopted as air duct and the WSHE is installed in the middle. An axial flow fan is installed at outlet to drive air flow. In addition, the air duct, water and the rubber tubes connecting the thermostatic bath with WSHE are wrapped with heat insulation foam to reduce heat loss.

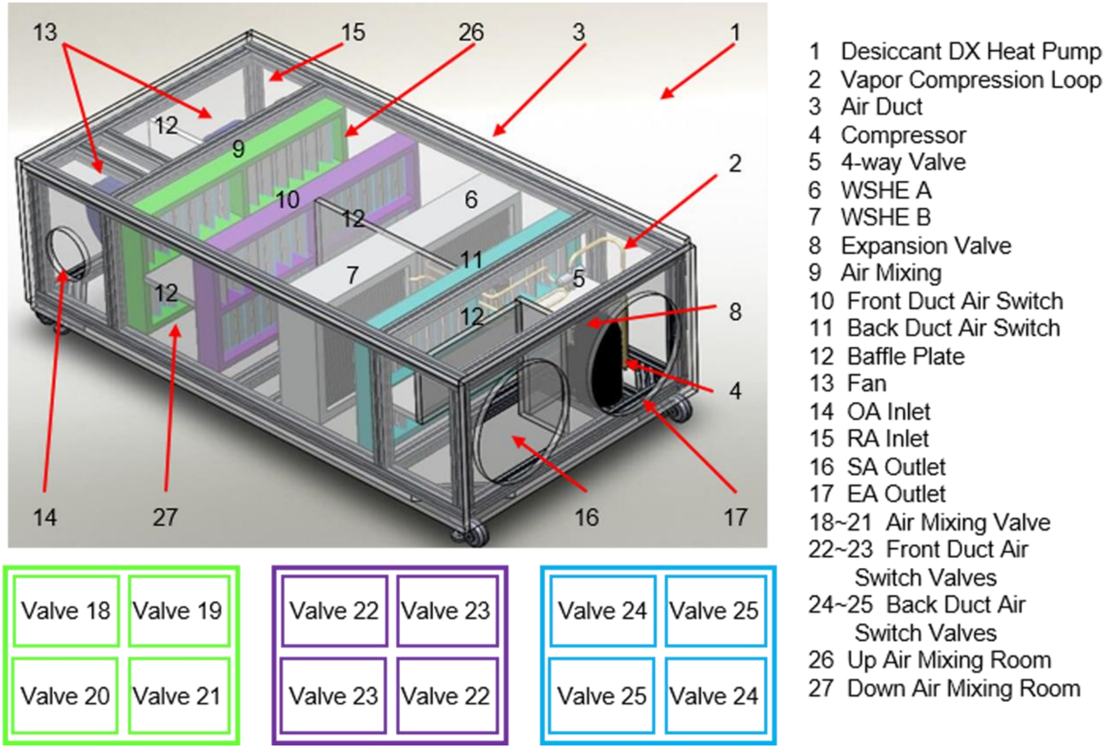

Fig. 4 Schematic diagram of novel design of desiccant-enhanced DX heat pump (DDX HP). DDX HP has two operation modes for cooling and dehumidification. (1) 4-way valve 5 were power-off, valve 22 and 25 are open and valve 23 and 24 are closed. WSHE 6 is condenser and WSHE 7 is evaporator. Outdoor air (OA) and return air (RA) are sucked into the cabinet by two fans 13 respectively. Part of OA passing through valve 18 and part of RA passing through valve 19 are mixed in chamber 26, formatting the process air; while the rest of OA passing through valve 20 and the rest of RA passing through are mixed in chamber 27, formatting the cooling air. Process air flows through valve 22(up) and then into WSHE 7; after being cooled and dehumidified, it passes valve 25(down) and at last is supplied into conditioned room by air duct 16. At the same time, cooling air flows through valve 22(down) and then into WSHE 6; after being heated and humidified, it passes valve 25(up) and finally is exited to the outdoor by air duct 17. (2) 4-way valve 5 are power-on, valve 22 and 25 are closed and valve 23 and 24 are open. WSHE 6 is evaporator and WSHE 7 is condenser. Process air flows through valve 23(up) and then into WSHE 6; after being cooled and dehumidified, it passes valve 24(down) and at last is supplied into conditioned room by air duct 16. At the same time, cooling air flows through valve 23(down) and then into WSHE 7; after being heated and humidified, it passes valve 24(up) and finally is exited to the outdoor by air duct 17.

Table 1. Parameters of WSHE used for characterization of airside heat and mass transfer

**Water-absorbing heat exchanger**	Outside diameter of copper tube	9.52mm
	Inner diameter of copper tube (smooth tube)	7.85mm
	Tube length	300mm
	Effective tube length	280mm
	Number of tube row	2
	Number of tube in each row	8
	Transverse tube pitch	25.0mm
	Longitudinal tube pitch	21.5mm
	Fin pitch	2.5mm
	Fin thickness (plain fin)	0.15mm
	Refrigerant flow path	one in-one out
	Airside heat transfer area	1.67m^2
	Frontal area	0.056m^2
	Base heat exchanger mass	0.9482kg
	Desiccant	CSGL
	Coated heat exchanger mass	1.2893kg
	Desiccant film thickness	~0.25mm

Table 2. Main parts used to construct the demo DDX HP.

	Type	Specifications	Manufacturer	Model
**Compressor**	Scroll	1.5HP	GMCC	DA130S1C-20FZ
**Throttle device**	EXV	500P	Sanhua Group	DPF(Q)2.0C-06-RK
**4-way valve**		2.0HP	Sanhua Group	SHF-9H-34U
**Water-absorbing heat exchanger**	Outside diameter of coppor tube			9.52mm
	Inner diameter of coppor tube (smooth tube)			7.85mm
	Tube length			340mm
	Effective tube length			320mm
	Number of tube row			4
	Number of tube in each row			12
	Transverse tube pitch			25.0mm
	Longitudinal tube pitch			21.5mm
	Fin pitch			2.5mm
	Fin thickness (plain fin)			0.15mm
	Refrigerant flow path			one in-one out
	Airside heat transfer area			5.72m^2
	Frontal area			0.096m^2
	Base heat exchanger mass			3.840kg
	Desiccant			CSGL
	Coated heat exchanger mass			5.675kg
	Desiccant film thickness			~0.25mm

Supplementary Note 1

Measurement Instruments

Two thermostatic baths are utilized to provide cold water and hot water, which have a volume of 30 L, control accuracy of ±0.05°C and volume flow rate of 15 $Lmin^{-1}$ by internal water pump. Two connected constant temperature and humidity chambers were adopted to simulate various outdoor and indoor air conditions. Each chamber has a space of 3m(length)×3m(width)×2.45m(height), which can supply constant air condition with temperature from -10 to 40°C, ±0.2°C accuracy, and relative humidity from 30% to 90%RH, ±5%*RH* accuracy. Temperature and humidity ratio of the air are measured by high accuracy and multi-functional digital Thermo/Hygrometer (type: TH110-PNA produced by KIMO Instruments). The measurement range of temperature is 20 to 80°C with accuracy of ±0.2°C and the measurement range of relative humidity is 0~100%RH with accuracy of ±1.7%RH. A thermoelectric anemometer (Kelong-VA40) is adopted to measure the air flow velocity and then the air flow rate can be calculated. Its measurement range and accuracy are 0~50ms^{-1} and ±0.015ms^{-1}. Other temperatures are measured by PT-100 RTD, with an accuracy of ±0.15°C. The mass flow rate of the water is measured by float meter. The measurement range is 60~600 Lhr^{-1} and the accuracy is ±4% of the measurement value. Suction pressure and discharge pressure of compressor were tracked by a high precision (±0.5%) pressure transmitter (MIK-P300), capable of measuring dynamic change between 0 and 6MPa. A coulomb meter (ZW3415B-RS) facilitates power consumption calculation. It can grasp power data from 0.2W to 10000W with error less than ±0.5%. All the measurement data are collected and transmitted to the recording computer by a data logger (Agilent-34970) with sampling time of 5s.

Data reduction

Water content of desiccant coat layer per unit mass Δw is used to investigate whether the dehumidification process could be viewed as an isothermal adsorption. Transient moisture uptake rate $\dot{w}$ is used to study the mechanism of water vapor uptake at different stages. Time-averaged moisture uptake rate $\overline{w}$ is used to simplify the temperature and humidity loosely-coupled control strategy. These parameters are defined as follows:

$$\Delta w = \sum_1^n \rho_a Q_a \left(d_{i,n} - d_{o,n}\right)\tau / m_s \quad (1)$$

$$\dot{w} = \rho_a Q_a \left(d_{i,n} - d_{o,n}\right) / m_s \quad (2)$$

$$\overline{\mathrm{w}} = \Delta w / (n\tau) \quad (3)$$

Where ρ_a is the density of moist air, Q_a is the air flow rate, n is the number of sampled data, τ is the data sampling period, m_s is the total mass of desiccant coat layer.

References

1. A. Freni, A. Frazzica, B. Dawoud, S. Chmielewski, L. Calabrese and L. Bonaccorsi, *Applied Thermal Engineering*, 2013, **50**, 1658-1663.
2. Y. Zhao, T. S. Ge, Y. J. Dai and R. Z. Wang, *Applied Thermal Engineering*, 2014, **63**, 52-58.
